# Laryngeal dystonia: case report and treatment with botulinum toxin

**DOI:** 10.1016/S1808-8694(15)30980-0

**Published:** 2015-10-19

**Authors:** Victor José Barbosa Santos, Fernando Marcos Mattioli, Wellerson Marcos Mattioli, Renata Jacob Daniel, Vicente Paulo Miranda Cruz

**Affiliations:** a Medical student; b MD – Medical School of the Federal University of Juiz de Fora; Residency in Otorhinolaryngology-Head and Neck Surgery - Hospital Servidor Público Estadual de São Paulo; Otorhinolaryngologist-Head and Neck Surgeon – Sinus Clinic, specialized in diseases of the ear, nose and larynx; c Medical Student - Fundação Educacional da Serra dos órgãos (FESO) - Teresópolis - RJ; d Voice specialist; Speech Therapist - Hospital nove de Julho de Juiz de Fora, MG; e Neurologist, Full member of the Brazilian Academy of Neurology, Professor of Neurology – Medical School - U.F.J.F., Full member of the Brazilian Academy of Neurology

**Keywords:** spasmodic dysphonia, laryngeal dystonia, botulinum toxin

## Abstract

Laryngeal dystonia or spasmodic dysphonia is characterized by involuntary and innapropiate spasms of vocal muscles, having the adductor type as the most common one. It is chacterized by strain-strangled voice with pitch breaks. Diagnosis is made by means of videolaryngostroboscopic exam. The treatment of choice is done with botulinum toxin directly injected in the muscles responsible for the mismatched movement. The aim of this study is to report on an adductor-type dysphonia patient and to discuss the advantages and observations about this treatment reported in the literature.

## INTRODUCTION

Laryngeal dystonia is a speech disorder characterized by involuntary contractions of the laryngeal muscles involved in the speech producing process. Although in the past they were treated as psychosomatic disorders, today we know that laryngeal dystonias or spasmodic dysphonias are central-motor processing neurological disorders. Koufman1 classifies laryngeal dystonias in four types (adductor, abductor, mixed and respiratory); the adductor type may be further divided in four subtypes depending on location (glottic, supraglottic) and the presence of tremor (with and without tremor); mixed dystonias are subdivided in three, based on the predominant disorder (adductor with abductor compensation, abductor with adductor compensation) and tremor. The adductor type is more frequent and characterized by tense and strangled voice, with frequent phonatory breaks and usually tremor due to irregularities and inadequate contractions of the muscles responsible for vocal cord adduction. In the glottic subtype, only those muscles involved in vocal fold adduction suffer this irregular contraction, and in the supraglottic, vocal folds, ventricular folds and other supraglottic structures are involved in the process, and speech fluency is most affected in this subtype. Abductor dystonia is less frequent and has worse results from Botulin Toxin treatment; it results from the lack of abductor muscles coordination and is clinically characterized by voice fluency breaks, with a breathy voice. Respiratory dystonia^2^ is a rare disease in which the irregular spasms do not occur during speech, but rather during inhaling, causing inspiratory stridor and inadequate respiratory pauses due to vocal folds paradoxical movements.

Diagnosis of the different dystonias is basically carried out by the following exams: general, otorhinolaryngological, neurological and stroboscopy. Laryngeal dystonias treatment is based on the injection of Botulin toxin directly in the abnormal muscle, identified through direct laryngoscopy or by percutaneous punction, monitored by flexible naso-laryngeal fibroscopy under local anesthesia and/or laryngeal electromyography^3^. Another injection may be necessary and according to some studies it should happen in approximately 6 months^4^, ^14^. Botulin toxin is produced by the Clostridiun tetani and it desensitizes the neural plaques where acetylcholine is used, thus inhibiting the spasms. It is yet to be defined if Botulin toxin may suffer resistance by the laryngeal muscles 6 and if repeated injections may cause their definitive desensitization, and if type B Botulin toxin may be used in cases resistant to type A^7^, ^10^. The goal of the present study is to report the case of a patient with laryngeal dystonia and discuss the treatment types mentioned in the literature.

## CASE REPORT

N. B., 61 year old Caucasian female, complaining of irregular speech breaks without hoarseness or aphonya. Symptoms started about 2 years ago, with voice loss after intense crying during many hours, and after some days her voice gradually returned, however with the aforementioned alterations. The patient went to a Neurologist and was diagnosed with dysarthria. She underwent speech therapy for six months, without changes in her vocal pattern.

She was assessed by the authors through stroboscopy, clinical neurological exam and voice lab. The patient presented signs of tense voice, vocal tiredness, breathy voice, laryngeal pain, loss in voice extension and lack of frequency control (oscillation). During examination she presented with mild hoarse voice, severe crepitation, having an inadequate voice pattern for her physique, with short maximum speech time for her gender and age. Negative response to auditory masking, thus ruling out the possibility of psychogenic dystonia, and she was diagnosed as having organic dysphonia due to laryngeal dystonia

Stroboscopy showed mild bilateral vocal tremor, more intense in the left vocal cord, no cord structural lesions, and no retrocrycoid and interarytenoid edema. She had good lamina propria expansion bilaterally without glottal gaps.

She was treated with the injection of 5U of type A Botulin Toxin (Botox) in the left thyroarytenoid muscle through suspension laryngoscopy. Her voice was somewhat breathy in the first week, and after that it became stable; she increased her respiratory capacity and maximum speech time, acquired respiratory pneumophonic coordination and better mobility of her phonoarticular organs. We then, decided to observe the patient and analyze her need for another injection of Botulin Toxin.

## DISCUSSION

Laryngeal dystonias are involuntary and non-coordinated contractions of the laryngeal muscles involved in speech production. They may be classified as adductors, abductors or respiratory, depending on the main muscles involved. Adductor dystonia is the most common^1^.

The case reported is about a patient with the adductor type laryngeal dystonia, and our discussion will emphasize this disorder.

The adductor dysphonia causes tense and strangled voice, with frequent speech breaks and intense vocal effort. It may be further divided into glottic and supraglottic adductor dysphonia, in the former only those muscles responsible for vocal cord adduction contract, while in the supraglottic type, the ventricular folds and supraglottic structures also play a role. They may be further classified according to the presence or not of tremor, and these are different from the essential tremors because they are not present during silence and calm breathing^1^.

Diagnosis is mainly clinical and may be helped by stroboscopy and electromyography, revealing irregular spasms of the adductor muscles during speech, increased and non-coordinated activity of the thyroarytenoid muscles in adductor dystonia.

Treatment is carried out through the intramuscular injection of Botulin toxin in the affected muscle groups, uni or bilateral and guided by endoscopy. Unilateral injection causes approximately the same effect, with less side effects8. The dose used is from 0.1U to 10U4. It has not been established whether repeated injections may cause resistance and if this resistance is caused by specific antibodies^9^. In the patients who develop Botulin toxin A resistance, the B type may be used^7^, ^10^. The therapeutic effect lasts for about 6 months, and the injection may be repeated after this period, depending on patient comfort and symptoms regarding his/her voice. Some studies used questionnaires to assess patient life quality changes after Botulin toxin injection, and it has proven to be very effective regardless of therapeutic time^4^, ^5^, it remains the treatment of choice for patients with laryngeal dystonia.

## CONCLUSION

The treatment of choice for spasmodic dystonias is the direct intramuscular injection of Botulin toxin and after about one week brings the patient's voice back to normal, causing comfort and speech fluency. Unilateral injection has the advantage of using a lesser dose, thus causing fewer side effects such as breathy voice on the days immediately after the injection, and it should be preferred in those cases in which stroboscopy and/or electromyography show greater contraction of a single vocal fold. The patient should be analyzed through stroboscopy and in the voice lab. Botulin toxin may be injected again depending on the results from these exams and patient's comfort and satisfaction.
